# Large anterior tibial artery pseudoaneurysm as a rare complication to vascular surgery

**DOI:** 10.1093/jscr/rjab303

**Published:** 2021-07-23

**Authors:** Emilie Nøddeskov Eilersen, Michael Strøm

**Affiliations:** Department of Vascular Surgery, Roskilde University Hospital, Roskilde, Denmark; Department of Vascular Surgery, Roskilde University Hospital, Roskilde, Denmark; Department of Vascular Surgery, Rigshospitalet, Copenhagen, Denmark; Copenhagen Academy for Medical Education and Simulation (CAMES), The Capital Region of Denmark, Copenhagen, Denmark

## Abstract

A pseudoaneurysm is a rare complication to vascular reconstruction and may be limb threatening if not treated. A patient previously treated for an aneurysm of the left popliteal artery presented to our outpatient clinic with swelling and reduced active movement. Computed Tomography Angiography revealed an 8.1 cm large pseudoaneurysm of the anterior tibial artery (ATA). The pseudoaneurysm was successfully treated with an interposition vascular graft to the patent ATA. Open surgical repair was the only option with resection of the pseudoaneurysm and insertion of an interposition vascular graft with and end-to-side anastomosis. The giant ATA pseudoaneurysm was successfully treated with insertion of a new end-to-side graft anastomosis with interposition to the old patent vascular graft.

## INTRODUCTION

A tibial artery pseudoaneurysm is a rare complication to vascular surgery and sparsely described in literature but may become limb threatening if not treated. Pseudoaneurysms are mainly reported following trauma [1–3] as well as an iatrogenic complication following surgery [4, 5]. The most commonly involved artery of the lower limb is the anterior tibial artery (ATA) [6]. A pseudoaneurysm can develop when all three layers of the arterial wall have been damaged. Blood penetrates from the artery into the surrounding tissue forming a fibrous capsule enclosing the blood flow outside the genuine lumen of the vessel. Treatment depends on the clinical presentation but may include external compression, ultrasound guided thrombin injection, endovascular stenting, embolization and open repair [3, 5, 7]. We present a case of a 68-year-old male with a large anastomotic ATA pseudoaneurysm which suddenly and rapidly developed several years after vascular bypass surgery treated with open resection and interposition graft surgery.

## CASE REPORT

A 68-year-old male was referred to our outpatient clinic with an increasing localized swelling and reduced movement of the lower left limb. The patient had a notable vascular history including aneurysms of the abdominal aorta and popliteal arteries. The right popliteal artery aneurysm had been prophylactically treated with a covered endovascular stent. The left popliteal artery had been prophylactically treated with femoral-popliteal *in situ* bypass surgery in 2004. The *in situ* bypass failed after 8 years leading to acute ischemia why the patient underwent secondary bypass surgery (GORE-TEX 7 mm ring reinforced, Flagstaff, Arizona) from the common femoral artery to the ATA, and amputation of the first toe. The patient was seen at our outpatient clinic 7 years and 4 months after the second surgery presenting a growing mass on the lateral side of the left leg developed over a period of 3 months. No fever, malaise or chills were reported. The patient had no recollection of recent leg trauma. Furthermore, no incisional infections after the first and second surgery were recorded.

**Figure 1:**
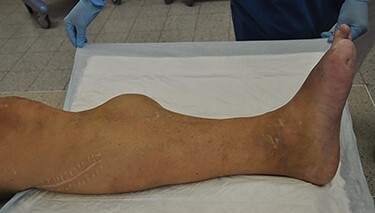
Indolent swelling of the left lower limb measuring 10 cm × 8 cm. Distal pulses were not palpable. Original magnification: 4024 × 2290 pixels.

**Figure 2:**
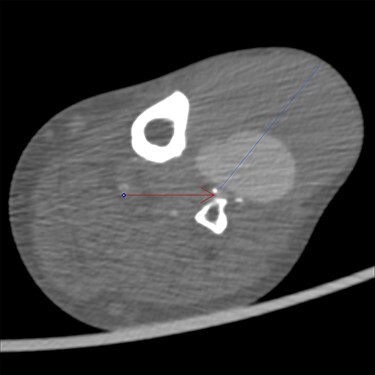
CTA showing the pseudoaneurysm at 75 mm (blue line) with both thrombus and contrast, and the patent anterior tibial artery (red arrow). Original magnification: 2000 × 2000 pixels.

Clinical examination revealed a 10 cm × 8 cm indolent swelling of the left lower limb (Fig. 1). Distal pulses were not palpable but distal blood pressure was 150 mmHg corresponding to ABI 0.77. Ultrasound revealed a 6.5 cm pseudoaneurysm of the ATA with a broad stem. Computed Tomography Angiography (CTA) showed that the reconstruction on the left leg was patent with a large 8.1 cm pseudoaneurysm of the ATA at the anastomosis (Fig. 2). Furthermore, the CTA revealed a 2 cm aneurysm of coeliac trunk, a 5.2 cm infrarenal abdominal aortic aneurysm, a 3.5 cm aneurysm of left distal superficial femoral artery (SFA) and a 6.7 cm occluded popliteal aneurysm.

**Figure 3:**
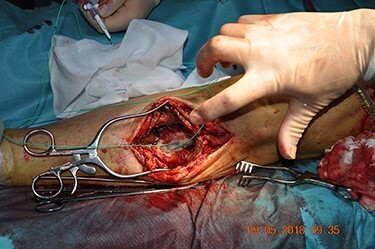
Temporary bleeding control ensured with Fogathy balloons in both ostia. Original magnification: 4288 × 2848 pixels.

**Figure 4:**
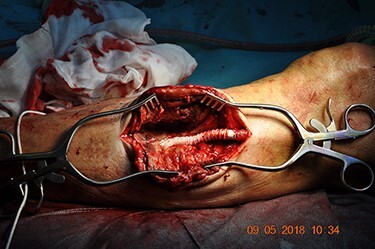
An 8 mm vascular graft anastomosed over the two ostia. Original magnification: 4288 × 2848 pixels.

The patient underwent open surgical exploration. A proximal incision over the left knee exposed the prosthesis for vascular control. The pseudoaneurysm was then exposed and the anastomosis was found to have disintegrated. The pseudoaneurysm was opened and the thrombus evacuated. The ostia of both the proximal and the distal ATA were visualized at the base of the aneurysm and found patent with reasonable back bleeding. Heparin was administered and temporary bleeding control was ensured with Fogathy balloons in both ostia (Fig. 3). An 8 mm vascular graft (GORE-TEX STRECTH ring reinforced, Flagstaff, Arizona) was anastomosed covering both ostia (Fig. 4). Five centimeters of the old graft were resected and anastomosed end-to-end to the new graft. The run-off was ensured with perioperative Doppler at the distal ATA.

Postoperatively the patient presented pulse in the dorsalis pedis artery and distal blood pressure of 185 mmHg corresponding to ABI 1.10. The patient showed no adverse effects from surgery and the reconstruction was patent at clinical examination 5 month postoperatively with no postoperatively signs of infection.

## DISCUSSION

Pseudoaneurysms of the crural arteries after vascular surgery are extremely rare and often linked to iatrogenic vessel injury [8, 9]. Different treatment regiments are suggested [10]. Endovascular intervention was deemed a nonviable option due to the large mismatch in diameter between the 7 mm graft and the genuine artery. Thrombin injection was not advisable due to the broad stem and risk of distal embolization to the only patent crural artery which could lead to leg ischemia and amputation.

We opted for open surgical exploration and repair with an end-to-side anastomosis covering both ostia and end-to-end anastomosis to the patent old graft to avoid a third operation in the groin. The distal part of the old graft had to be resected through a very fibrous field in order to expose the ostia of the native vessel. Control of bleeding was ensured with the balloons which also reduced surgical exploration in the very fibrous field. A bypass from above the knee was not possible as the SFA was aneurysmatic. The artery could have been explored further distally in order to perform an end-to-end anastomosis in fresh tissue with secondary ligation of the proximal artery. However, the old graft was completely segregated from the artery leaving a defect in the anterior wall and an acceptable posterior vessel wall. This allowed for the anastomosis to be performed end-to-side resulting in both antegrade and retrograde perfusion of the vessel.

A loose suture may allow blood to penetrate between the graft and the genuine artery with the development of a pseudoaneurysm. It may be assumed that the expanding pseudoaneurysm led to a total rupture of the anastomosis resulting in a large pseudoaneurysm that functioned as segue from the graft to the patent ATA.

While anastomotic pseudoaneurysms are a known risk following graft surgery, it is surprising that the pseudoaneurysm developed more than 7 years after surgery.

The patient was not tested for a connective tissue disorder which would be beneficial given the patient’s multiple aneurysms. The pseudoaneurysm was not investigated for infectious ethology since the patient showed no clinical or para-clinical signs of graft infection and the CTA revealed a delimited pseudoaneurysm with no radiological signs of infection or mycotic process. The patient showed increased CRP and blood count after surgery corresponding to an expected postoperative response. In June 2021 a competing systemic infection led to PET-CT which showed no signs of infection of the prosthetic bypass graft or the left lower limb. The aneurysm of the SFA was not treated since the patient revealed no related symptoms and ligation should be performed either through high thigh incision or through a severely scarred groin.

In conclusion, pseudoaneurysms of the tibial arteries after open revascularization are extremely rare. We presented a case of a very late onset giant pseudoaneurysm of the ATA 7 years after vascular graft surgery which was successfully treated with open surgical repair.

**Highlights**

- Large pseudoaneurysm successfully treated with open surgical repair
  Pseudaneurysm of the anterior tibial artery is rare following vascular surgery
- Consensus regarding treatment is lacking
- Psuedoaneurysm of the anterior tibial artery may be limb threatening if not treated
